# Optimal transport- and kernel-based early detection of mild cognitive impairment patients based on magnetic resonance and positron emission tomography images

**DOI:** 10.1186/s13195-021-00915-3

**Published:** 2022-01-07

**Authors:** Ziyu Liu, Travis S. Johnson, Wei Shao, Min Zhang, Jie Zhang, Kun Huang

**Affiliations:** 1 Department of Statistics, Purdue University, West Lafayette, USA; 2 Department of Biostatistics and Health Data Science, Indiana University School of Medicine, Indianapolis, USA; 3 Department of Medical and Molecular Genetics, Indiana University School of Medicine, Indianapolis, USA; 4 Regenstrief Institute, Indianapolis, USA

**Keywords:** Transfer learning, Optimal transport, Bootstrap aggregation

## Abstract

**Background:**

To help clinicians provide timely treatment and delay disease progression, it is crucial to identify dementia patients during the mild cognitive impairment (MCI) stage and stratify these MCI patients into early and late MCI stages before they progress to Alzheimer’s disease (AD). In the process of diagnosing MCI and AD in living patients, brain scans are collected using neuroimaging technologies such as computed tomography (CT), magnetic resonance imaging (MRI), or positron emission tomography (PET). These brain scans measure the volume and molecular activity within the brain resulting in a very promising avenue to diagnose patients early in a minimally invasive manner.

**Methods:**

We have developed an optimal transport based transfer learning model to discriminate between early and late MCI. Combing this transfer learning model with bootstrap aggregation strategy, we overcome the overfitting problem and improve model stability and prediction accuracy.

**Results:**

With the transfer learning methods that we have developed, we outperform the current state of the art MCI stage classification frameworks and show that it is crucial to leverage Alzheimer’s disease and normal control subjects to accurately predict early and late stage cognitive impairment.

**Conclusions:**

Our method is the current state of the art based on benchmark comparisons. This method is a necessary technological stepping stone to widespread clinical usage of MRI-based early detection of AD.

**Supplementary Information:**

The online version contains supplementary material available at (10.1186/s13195-021-00915-3).

## Background

AD is an irreversible, degenerative brain disorder, affecting over six million Americans and is the sixth leading cause of death in the USA [1]. AD is hallmarked by neuron loss [2], inflammation [3], amyloid plaques [4], and tau deposition [5], which lead to progressive tissue loss in the brain and cognitive decline in the patient [6]. Diagnosing AD is largely based on tests of cognitive impairment combined with technologies such as CT, MRI, or PET but can only be verified after death on the postmortem brain [7]. Patients who have not yet progressed to AD may be diagnosed with mild cognitive impairment (MCI). The direct definition of MCI has undergone recent changes. Due to these changes, the diagnostic quality of the MCI designation is only becoming more well refined with improved research into patient stratification [8] and diagnostic guidelines [9]. Despite the fact that MCI is not a prodromal stage of AD, it is a transitional phase between normal cognitive aging and AD in which individuals demonstrate objective cognitive impairment and report subjective complaints but have relatively intact functional abilities [8]. Since early and late stage MCI (E-MCI and L-MCI) have different survival rates and rates of developing AD [9], to help clinicians to provide timely treatment and delay disease progression, it is crucial to identify E-MCI and L-MCI patients with higher rates of biomarker abnormalities and progression to AD [8]. In the process of diagnosing MCI and AD in living patients, brain scans are collected using neuroimaging technologies such as CT, MRI, and PET to rule out other potential causes of the disease. These brain scans measure the volume and molecular activity within the brain resulting in a very promising avenue to diagnose patients.

Specifically, neuroimaging techniques enable us to identify regions of interests related to AD [10] and extract sensitive markers for AD. It has been demonstrated that features extracted from structural MRI and PET can help us investigate the neurophysiological feature of AD and MCI [11, 12]. These features can be utilized to diagnose the early stage of AD patients and predict whether an MCI patient will progress to AD [13]. We seek to utilize these features for distinguishing E-MCI versus L-MCI and formulate this problem as a classification task.

Recent progress in machine learning (ML) and pattern recognition methods shed light on the diagnosis of AD patients with the help of neuroimaging features. Despite the wide applications of ML models in biomedical problems, there are two major challenges in classifying MCI stages, namely that the collection of multiple-modality datasets is costly and time consuming, and that the effect size observed between E-MCI and L-MCI is too small compared with the feature dimension. This may lead to the overfitting issue, which occurs when the model performs well on training samples while lacks generalizability on unseen data. We seek to enlarge the training sample size to overcome the overfitting problem and improve model stability.

Accordingly, it is of great interest to develop ML models that utilize samples from easier-to-train tasks that are related and have more readily available data. In dementia, the AD patient versus NC patient task can be leveraged to transfer knowledge to the more challenging task of predicting MCI stage. Some previous works [13, 14] introduced auxiliary tasks such as the AD and NC classification task to identify disease related features and construct the decision function for classification. Transferring knowledge from different but related auxiliary tasks to increase the prediction accuracy on a more difficult target task is a widely used ML strategy called transfer learning (TL). TL uses heterogeneous data and has to face the challenging ML dilemma as the decision function learned from the source (auxiliary) task cannot be directly applied to the target domain. Two heterogeneous datasets will occupy different distributions in the feature spaces, which is termed distributional drift. Traditional TL techniques adopt sample weighting strategies and feature alignment strategies [15] to overcome the distributional drifting problem. Recently, Optimal Transport (OT) theory has been successfully introduced in TL problems [16, 17]. Since OT has shown great promise in tackling the data drifting (target shifting) issue, we adopt it in our model to address the difficulty of utilizing AD and NC samples for tackling our L-MCI and E-MCI stratification problem.

Our model consists of three main components: feature selection, TL, and bootstrap aggregation. We will first use the robust multi-label transfer feature learning rMLTFL [13] framework, which can be used for feature selection as well as the traditional one-way ANOVA to select representative features from MRI and PET data modalities. Then, we will develop the OT TL strategies to train classifiers for stratifying L-MCI and E-MCI with the help of AD and NC samples. Finally, we will apply the Bootstrap Aggregation (BAg) strategy to overcome the overfitting problem and improve stability and accuracy.

## Methods

### Data collection and preprocessing

The Alzheimer’s Disease Neuroimaging Initiative (ADNI) provides researchers with multi-modal longitudinal data for subjects as they work to define the progression of AD. The ADNI-1 dataset contains 202 subjects with MRI and PET brain images. The updated dataset ADNI-2 assessed participants from the ADNI-1 phase besides new participant groups including elderly controls and subjects with significant memory concern, E-MCI, and L-MCI. We summarize the samples used in our study in Table 1.

**Table 1:** The values are expressed as mean ± standard deviation

	NC	E-MCI	L-MCI	AD
Number	211	273	187	160
Gender (M/F)	190/101	153/119	108/76	95/65
Age	76.1 ±6.5	71.5 ±7.1	73.9 ±8.4	75.2 ±7.9
Education	16.4 ±2.6	16.1 ±2.6	16.4 ±2.8	15.9 ±2.8
MMSE	29.0 ±1.2	28.4 ±1.5	27.7 ±1.7	24.0 ±2.6
CDR	0.0 ±0.1	0.5 ±0.1	0.5 ±0.1	0.7 ±0.3

*AD* Alzheimer’s disease, *NC* normal control, *E-MCI* early mild cognitive impairment, *L-MCI* late mild cognitive impairment, *MMSE* Mini-Mental State Examination, and *CDR* clinical dementia rating

The feature extraction process includes image registration, region of interests selection, and feature quantification. We specifically use the morphometry features extracted from voxel-based measures of structural MRI (VBM-MRI) and fluorodeoxyglucose positron emission tomography (FDG-PET) images and denote the two classes of features as VBM and FDG features (Additional files 1 and 2). The details of feature extraction can be found in the Materials and workflow section of [18].

### Feature selection

To reasonably utilize informative features from the two data modalities, we used the robust multi-label transfer feature learning (rMLTFL) model [13] to filter out features that are irrelevant to the classification task. In the study by Cheng et al. [13], this model was applied to select features to train a support vector machine (SVM) model for distinguishing Progressive MCI (P-MCI) and Stable MCI (S-MCI). This framework can help identify features related to the target task (L-MCI vs E-MCI) that benefit from auxiliary tasks (AD vs NC, AD vs MCI, MCI vs NC). However, it faces a difficult situation that separating E-MCI and L-MCI samples using linear SVM and logistic regression (LR) is not effective, even with multiple kernels. Therefore, we only adopted it as a feature selection method and compared it with the traditional one-way analysis of variance (ANOVA) feature selection technique.

We denote the dataset on the target task (L-MCI vs E-MCI) as (**X**^1^,**X**^2^,**y**^*t*^). $\mathbf {X}^{1}, \mathbf {X}^{2} \in \mathbb {R}^{460 \times 116}$ represent the FDG and VBM features respectively while **y**^*t*^∈{−1,+1} is the class label correspond to E-MCI and L-MCI respectively. We also construct three auxiliary domains $\{(\mathbf {A}^{1}_{1}, \mathbf {A}^{2}_{1}, \mathbf {y}^{a}_{1}), (\mathbf {A}^{1}_{2}, \mathbf {A}^{2}_{2}, \mathbf {y}^{a}_{2}), (\mathbf {A}^{1}_{3}, \mathbf {A}^{2}_{3}, \mathbf {y}^{a}_{3})\}$. Each triplet in the bracket represents a task that may be helpful for feature selection. For instance, $(\mathbf {A}^{1}_{2}, \mathbf {A}^{2}_{2}, \mathbf {y}^{a}_{2})$ denotes the FDG and VBM features along with labels for AD (+1) and NC (-1) patients. To construct a *multi-bit label coding matrix* for the TL task, we firstly trained three logistic regression models on three auxiliary domains. Then, we used these three models to independently estimate three labels for each patient on the target domain. Finally, we concatenated the true label with three predicted labels to form a multi-bit label for each patient and obtain a multi-bit label matrix $\mathbf {Y} = [\mathbf {y}^{t}, \mathbf {y}^{p}_{1}, \mathbf {y}^{p}_{2}, \mathbf {y}^{p}_{3}]\in \mathbb {R}^{460 \times 4}$ (one true label, three predictions). The goal of the rMLTFL algorithms is to learn a weight matrix $\mathbf {W} = [\mathbf {w}^{t}, \mathbf {w}_{1}, \mathbf {w}_{2}, \mathbf {w}_{3}] \in \mathbb {R}^{116 \times 4}$ which can be decomposed into two components **P** and **Q** for feature selection and domain identification respectively. Specifically, the objective function is formulated a following:

1 $$ \begin{aligned} \min_{\mathbf{W}, \mathbf{P}, \mathbf{Q}} \left\Vert \mathbf{Y} - \mathbf{X}\mathbf{W}\right\Vert_{F}^{2} + \lambda_{1} \left\Vert \mathbf{P} \right\Vert_{2, 1} + \lambda_{2} \left\Vert \mathbf{Q}^{T} \right\Vert_{2, 1} + \\ \lambda_{3} \sum\limits_{i=1}^{3} \left\Vert (\mathbf{X}\mathbf{w}^{t} - \mathbf{X}\mathbf{w}_{i}) - (\mathbf{y}^{t} - \mathbf{y}^{p}_{i})\right\Vert_{2}^{2}, \\ s.t.\ \mathbf{W} = \mathbf{P} + \mathbf{Q} \end{aligned}  $$

The first term is to ensure the similarity between the multi-bit labels **Y** and its prediction **XW**. In the second and the third term, we use the 2,1 norm to capture the shared features across all tasks and filter out the unrelated tasks. The 2,1 norm forces some rows of **P** and some columns of **Q** to be all zero. Non-zeros rows in **P** and non-zero columns in **Q** corresponds to informative features and tasks respectively. The last term indicates that the distance from predicted target domain label **Xw**^*t*^ to multi-bit label $\mathbf {X}\mathbf {w}^{p}_{i}$ should be similar to the distance from the true label **y**^*t*^ to the estimated multi-bit label $\mathbf {y}^{p}_{i}$.

**Fig. 1:**
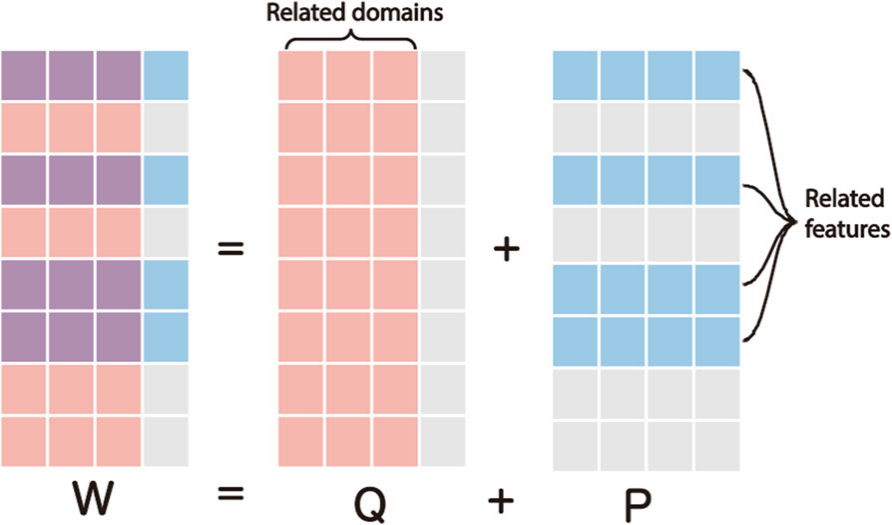
The learnable weight matrix **W** can be decomposed into two matrices, **Q** and **P**. They are responsible for selecting target problem related tasks (AD vs NC, AD vs MCI, MCI vs NC) and features. By enforcing the *l*2−*l*1 norm of **Q**^*T*^ and **P** to be small, these group lasso penalty terms on rows on **P** and columns of **Q** encourage the rows of **P** and columns of **Q** in (1) to have all zero (rows and columns in gray) or non-zero elements. The first column of **Q** corresponds to the L-MCI vs E-MCI stratification task and the rest of them correspond to three auxiliary tasks. We could observe from the plot that the AD v NC and the AD vs MCI tasks are two related domains while the MCI vs NC task could not provide helpful information. Similarly, non-zero rows of **P** capture the shared features among useful domains

The above rMLTFL framework to select feature can be illustrated in Fig. 1. After we obtained the multi-bit label matrix **Y**, we used the accelerate gradient descent algorithm to optimize the target function (1). Then, we filtered out domains that corresponded to all zero columns in **Q**. After that, we repeated the same process as above without these useless domains. Finally, we selected rows that corresponded to non-zero rows in **P** as features related to the target task. When implementing rMLTFL and one-way ANOVA to select features, we applied each method to the two data modalities separately and simultaneously. Hence, we obtained six sets of sample features. After examining the prediction performance of these feature sets, we chose the most relevant feature sets and achieved higher prediction accuracy by applying model aggregation techniques.

### Optimal transport for transfer learning

In previous work of MCI stage classification, i.e., classifying P-MCI vs S-MCI [13] and MCI converters versus MCI non-converters [14], a common assumption is that introducing auxiliary tasks (ie. AD vs NC) can improve the accuracy of classification. It is assumed that at least some of these auxiliary domains can help us understand the target domain, even without feature transformation. From the t-distributed stochastic neighbor embedding (t-SNE), boxplot of principal components, and violin plot of features we conclude that the feature distribution of L-MCI and E-MCI is similar to the pattern of those in the AD and NC subjects. However, the difference between early and late stage MCI is much more subtle than the difference between AD and NC samples. Therefore, we must adopt TL strategies to reduce the inter-task discrepancy between AD vs NC task and E-MCI vs L-MCI task while maximizing the intra-task differences. Traditional TL methods using sample weighting or feature alignment strategies to adapt source data samples (i.e. AD and NC samples) to the target domain (i.e., L-MCI and E-MCI samples)[15]. Compared with these previous works, the OT for TL frameworks can capture the intrinsic geometry structure difference of two feature spaces and address the distributional drift problem more efficiently. We illustrate in our experiments that our proposed method based on OT outperforms the current state-of-the-art methods.

OT maps samples from one domain to another by minimizing the earth mover’s distance [16, 19] between sample distributions in two domains. To better understand the feature distribution within and across classes and to estimate a better transformation, [16, 17, 20] added different regularization terms such as *L*_1_*l*_2_ and *L*_*p*_*l*_1_ terms to achieve group sparsity. By adding the group sparsity regularization terms, the OT feature mapping strategy only projects L-MCI training samples to the AD samples and E-MCI training samples to the NC samples. For computational efficiency, most of the state-of-the-art OT models incorporate an entropy regularization term. This regularized version of earth mover’s distance [21] is call Sinkhorn distance (SD). In this study, we implemented three OT mapping strategies defined by SD, SD with *L*_*p*_*l*_1_ regularization term, and SD with *L*_1_*l*_2_ regularization term respectively.

**Fig. 2:**
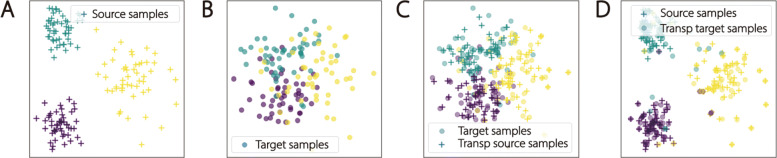
We use a synthetic Gaussian distributed dataset to demonstrate our method. In panel **A**, we generate three clusters of gaussian distributed samples. Their clusters are distinct, hence simple decision boundaries can separate them clearly. This example corresponds to the AD vs NC classification task. In panel **B**, we also generate three clusters which are not distinctive from one another. In fact, the E-MCI and L-MCI clusters are much less distinct than the samples in panel **B**. In panel **C**, we use OT to map the source domain samples onto the target domain. In the last panel (**D**), we use our proposed method adopting OT to map target samples onto the source domain by utilizing sample labels

Before introducing the experiment setting of using OT to train classifiers, we want to emphasize the difference between our proposed method and traditional OT methods for TL that are used as benchmarks in this study. Traditionally, the source domain (AD vs NC) features are mapped to the target domain (L-MCI vs E-MCI) via an OT strategy. Then, AD and NC labels as well as the transformed features can be used to train a classifier on the target domain that will be directly applied to the L-MCI vs E-MCI stratification task. This strategy is powerful when dealing with the condition that few labels are available on the target domain and the decision boundary for the target task is easy to learn. In our problem, the intrinsic difficulty is that the decision boundary is difficult to learn even after using kernel methods. Fortunately, we have plenty of samples (187 L-MCI, 273 E-MCI) on the target domain, which enable us to separate them into training and testing sets. Therefore we instead map training samples on the target domain(L-MCI vs E-MCI) to the source domain (AD vs NC) where the classification boundary is more clearly defined. During this process, we learn a non-linear OT mapping strategy **T**. Then, we train classifiers to use AD and NC samples as well as E-MCI and L-MCI samples transformed by **T**. After that, we use the OT mapping **T** to project testing samples to the source domain and use the classifier to stratify E-MCI and L-MCI samples. Finally, we evaluate the classification performance using accuracy and area under the receiver operating curve (AUC) score. Figure 2 illustrates the effects of using OT to obtain more distinguishable features in synthesized data.

**Fig. 3:**
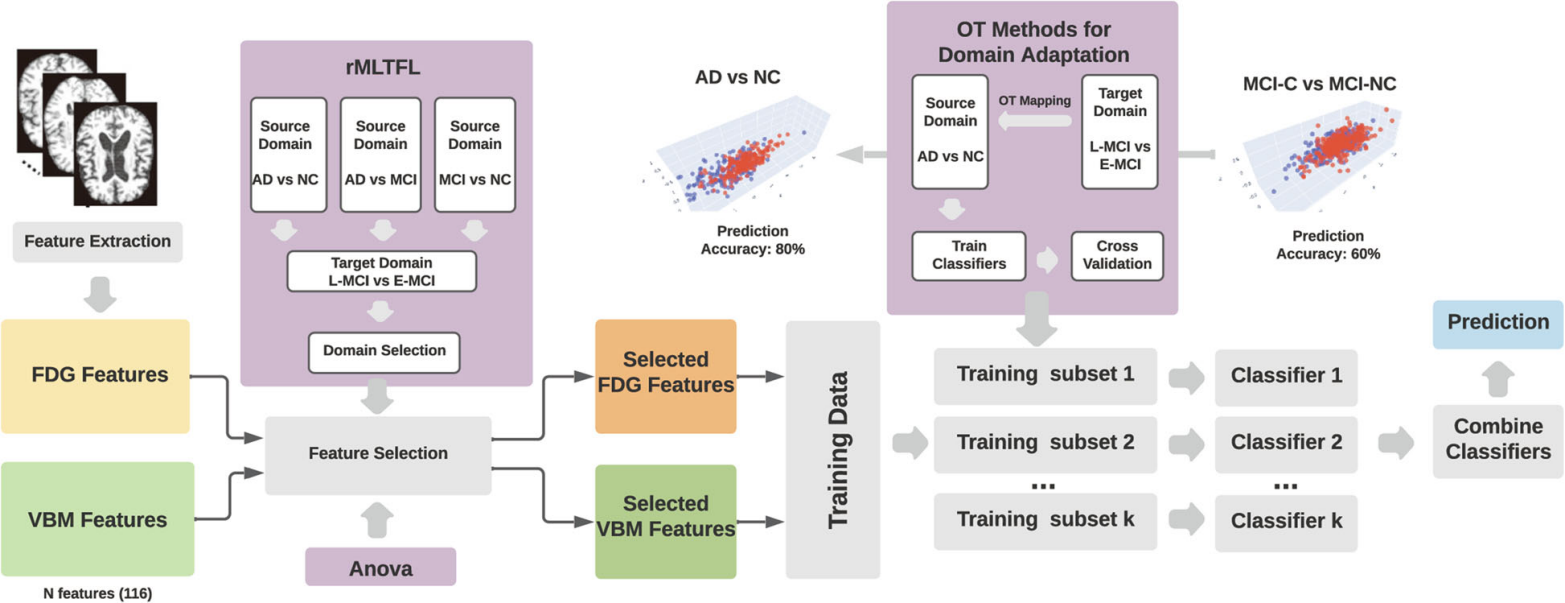
Our novel OT TL framework and pipeline were used to train the patient stratification model. Using the pre-processing workflow, we extract VBM and FDG features from the manually labeled regions-of-interest (ROIs) in MRI and FDG images respectively. Then, we use the rMLTFL framework as well as one-way ANOVA to select features from two modalities (FDG and VBM) both separately and simultaneously. We then separate the target dataset into training (80%) and testing (20%) sets. After that, we sample subsets of training samples, use the regularized OT to mapping selected samples to the AD vs NC data domain, and train classifiers using labeled AD, NC, and transformed samples. Finally, we aggregate these models to form a robust BAg model and make predictions on OT transformed testing samples

In our experiments on real AD data, we investigate different OT mapping strategies as well as different classifiers on the source domain. In Fig. 3, we illustrate how to adapt MCI samples onto the AD and NC domain. In Fig. 5 panel (**A**), we demonstrate how to combine different OT mapping strategies with different classifiers. Since logistic regression achieves higher prediction accuracy than SVM, we adopt it as a benchmark classifier and combine it with linear and polynomial kernel functions to form kernel based classifiers.

### Bootstrap aggregation to improve model stability

Bootstrap aggregation (BAg) is an algorithm proposed in [22] for both regression and statistical classification. By randomly sampling training sets (bootstrapping) with replacement, one can train several classifiers using the same algorithm. By aggregating model predictions based on the majority voting strategy or aggregating prediction probabilities, we raise the stability of our models by reducing inter-model variability from overfitting. When we implemented the BAg strategy, we first need to decide the number of “bags” to use. Since our study only contained a few hundreds samples, We used 5 bags to train five sub-models. Then, we aggregated the model using a majority vote strategy. The prediction probability was obtained by calculating the mean prediction probability across each sub-model. We illustrated the pipeline in Fig. 3.

### Feature selection comparison

Using one-way ANOVA, we calculated the *p* value for each feature individually. Using the *p* value threshold 0.05, we selected 47 out of 116 features from the FDG and the VBM data modality respectively. The rMLTFL method captures features by training a model and selecting features based on that trained model. We need to verify the stability of this feature selection procedure. To determine which hyper-parameters to use and whether the collection of useful features were dependent on the training set, we used five-fold cross-validation to verify the robustness of the rMLTFL method. We took a grid search approach for the three hyper-parameters over 1000 combinations of these parameters and chose the hyper-parameter combination with the highest average prediction accuracy. Using the optimal hyper-parameters, we ran the rMLTFL algorithm on the FDG data modality to filter out useless features and obtained 96 features by merging selected features respectively across five folds. For the VBM data modality, the model only filtered out one useless features over all hyper-parameter combinations. Therefore, we kept 115 feature from the VBM data modality. To combine the two data modalities, we concatenated the two feature vectors and repeated the same process as described above. We visualized the selected features ability to stratify NC, E-MCI, L-MCI, and AD individually and aggregated via PCA and tSNE plots.

### Transfer learning benchmark comparison

First we benchmarked different OT mapping strategies via ten-fold cross-validation on each data modality individually and the combined data modalities. Specifically, we applied three different OT mapping regularization strategies: SD (OT regularized by an entropy regularization term), SD regularized by *L*_*p*_*l*_1_ norm, and SD regularized by *L*_1_*l*_2_ norm to map samples from the target domain to the source domain. The usage of these regularization norms is to enforce intra-class similarity and improve computational efficiency. When we mapped L-MCI and E-MCI samples to the domain of AD and NC samples, we utilized the labels of training samples, i.e. E-MCI and L-MCI. Using these transformed samples as well as AD and NC samples, we evaluated the performance on the source domain via accuracy and AUC scores.

Besides two baseline methods and the rMLTFL framework, we also compared our model with other TL benchmarks and multiple kernel learning strategies. For TL benchmarks, we compared our method with: Importance-weighting with logistic discrimination (IW) [23], Transfer Component Analysis (TCA) [24], Semi-supervised Subspace Alignment (SUBA) [25], Feature-Level Domain Adaptation (FLDA) [26], and Boosting for Transfer learning (TrAdaBoost)[27]. We also compared with multiple kernel learning strategies including: the simple average of base kernels (AverageMKL), margin-based combination of kernels (EasyMKL) [28], radius-margin ratio optimization for dot-product boolean kernel learning (GRAM) [29], margin and radius based multiple kernel learning (RMKL) [30], simple but effective methods for combining kernels in computational biology (PWMK) [31], and centered kernel alignment optimization in closed form (CKA) [32]. Since we use the decision tree as a basic classifier for some of these benchmarks, e.g. TrAdaBoost, we can’t obtain the AUC score directly. To evaluate the model performance, we use ten-fold cross-validation and calculate the average and standard deviation of the accuracy score.

### Bootstrap aggregation comparison

We separated the dataset into training and testing sets (80% and 20%). On the training set, we implemented the bootstrap strategy in a slightly different manner. During the stage of bootstrapping, we randomly split the training set into five folds and picked four folds each time to train a classifier using our OT TL strategy. To demonstrate that our OT alignment improves the stratification performance, we also compared our method with different versions of BAg using traditional SVM, logistic regression, and rMLTFL models as classifiers.

## Results

### Diagnostic value of MRI features

**Fig. 4:**
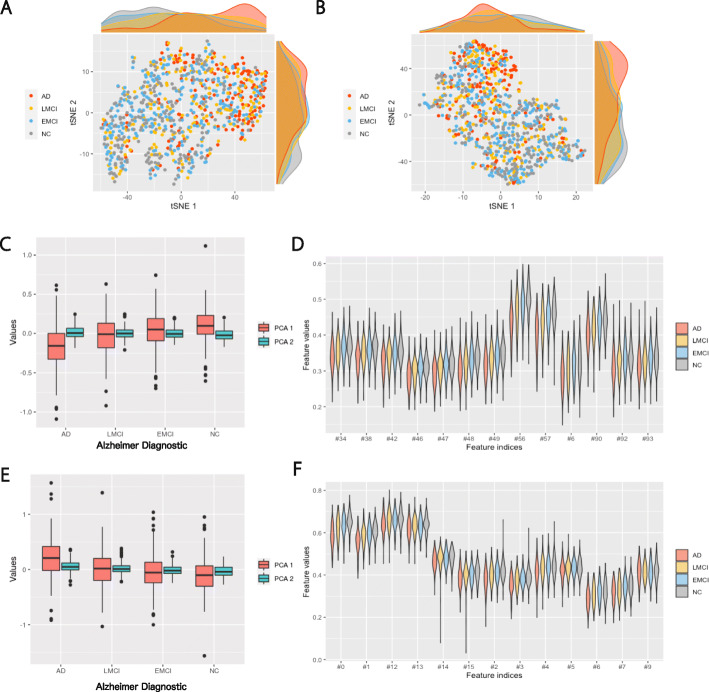
**A** and **B** represent t-SNE plots and their marginal distributions for **VBM** features selected by ANOVA and rMLTFL respectively. **C** and **E** are box-plots for first two principle components of these selected features. We also visualize the distribution of feature contributions (input feature values after preprocessing to [0,1] scale) selected by ANOVA (**D**) and rMLTFL (**F**)

We visualized the selected VBM features in Fig. 4. Panels **A** and **B** show the t-SNE plots of features selected by ANOVA and rMLTFL respectively. In panel **A**, we observed that AD patients mainly concentrated on the upper right corner where L-MCI patient is also denser than other areas while E-MCL and NC samples are denser at the lower left corner. We concluded that the pattern of AD vs NC may help us delineate the distributions of L-MCI versus E-MCI. The same pattern can be observed in panel **B**. Panel **C** and **E** illustrate distributions of first two principle components of ANOVA and rMLTFL features. From these plots we concluded that the distributional differences between the first principle components of L-MCI and E-MCI patients are more subtle than the differences between AD and NC patients. AD and L-MCI patients tended to have lower PC 1 while E-MCI and NC tend to have higher values of PC 1. We also visualized part of features selected by ANOVA and rMLTFL in **D** and **F**. From them we observed the same pattern as the boxplots.

### Transfer learning benchmark comparison results

The results of cross-validation for FDG and VBM data modalities in Tables 2 and 3 demonstrate that our framework outperformed all baseline methods and the original rMLTFL model by increasing prediction accuracy and reducing variability. Based on FDG features, our model achieved 68.76±7.53*%* accuracy and 0.66±0.08 AUC score across ten-fold cross-validation. The SVM and logistic regression baseline methods achieved 61.20±7.22*%* and 64.40±7.60*%* accuracy respectively. Our model also outperformed them on the VBM data modality. Comparing the performance of features selected by rMLTFL and ANOVA, we observed that the rMLTFL features are always superior than ANOVA features. This indicates that even features that are not significant statistically may be helpful to model complex nonlinear differences between sample classes. Combining two data modalities by directly concatenating features did not help us in distinguishing L-MCI and E-MCI patients.

**Table 2:** Accuracy (ACC) and AUC score of models based on features selected by rMLTFL and ANOVA (*p* value threshold=0.05) respectively

		Sinkhorn distance		Sinkhorn distance + *L*_1_*l*_2_		Sinkhorn distance + *L*_*p*_*l*_1_	
		ACC	AUC	ACC	AUC	ACC	AUC
FDG	rMLTFL	68.76 ±7.53	0.66 ±0.08	66.04 ±7.53	0.65 ±0.08	65.48 ±5.04	0.64 ±0.07
	ANOVA	66.07 ±6.96	0.65 ±0.07	63.63 ±6.01	0.64 ±0.07	59.50 ±6.53	0.62 ±0.08
VBM	rMLTFL	62.37 ±6.88	0.62 ±0.11	62.74 ±0.08	0.62 ±0.11	57.86 ±6.32	0.60 ±0.07
	ANOVA	58.94 ±7.82	0.59 ±0.12	58.68 ±0.08	0.58 ±0.12	56.79 ±0.11	0.58 ±0.13
FDG + VBM	rMLTFL	62.26 ±6.48	0.63 ±0.05	66.61 ±6.29	0.65 ±0.06	66.05 ±5.91	0.65 ±0.08
	ANOVA	61.44 ±6.23	0.64 ±0.05	63.87 ±5.75	0.63 ±0.06	61.15 ±8.01	0.64 ±0.07

**Table 3:** Accuracy of baseline, transfer learning and Multi-kernel benchmark methods

Methods	FDG	VBM
SVM	61.20 ±7.22	57.64 ±5.89
Logistic Reg	64.40 ±7.60	58.72 ±6.98
rMLTFL	63.33 ±9.02	62.53 ±9.08
IW	60.10 ±8.41	59.56 ±7.49
TCA	59.83 ±6.02	57.02 ±8.27
SUBA	64.68 ±4.34	52.44 ±8.33
RBA	61.46 ±8.21	58.17 ±8.02
FLDA	63.90 ±10.00	60.11 ±9.05
TrAdaBoost	61.45 ±8.56	58.98 ±7.43
Easy MKL	64.72 ±9.75	60.38 ±7.46
Average MKL	63.34 ±9.08	60.11 ±7.14
PWMK	64.19 ±9.80	60.11 ±7.14
GRAM	64.72 ±9.75	/
RMKL	63.91 ±9.53	60.11 ±7.14
CKA	59.56 ±7.49	59.56 ±7.49

The values are denoted as mean ±standard deviation

**Fig. 5:**
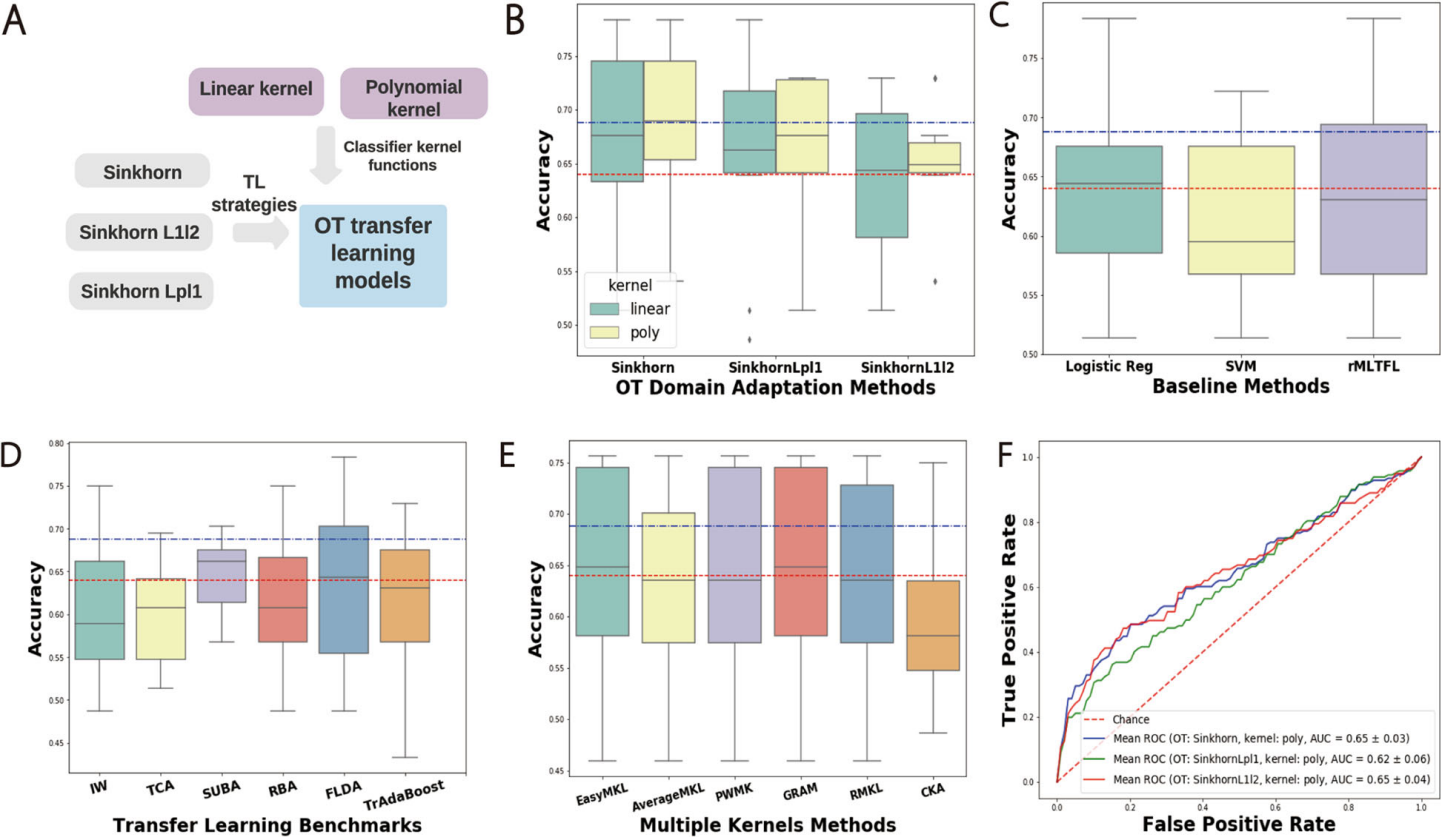
Results of ten-fold cross-validation using our method and other benchmark methods on FDG features. Panel **A** is the working pipeline of our OT TL model. We combine linear and polynomial kernelized logistic regression classifier with different OT mapping strategies. In **B**, we represent the accuracy score of different OT and kernel combinations. The blue and red horizontal lines represent the average accuracy of our best model and the logistic regression model respectively. In panel **C**, we demonstrate the performance of two baseline methods, e.g. logistic regression and SVM, and the rMLTFL model. In **D** and **E**, we visualize the performance of TL benchmarks and Multi-kernel learning strategies. In **F**, we plot the AUC curve of our model across ten folds

Based on the TL benchmark experiments, our method proved superior to all of these benchmarks (Table 3, Fig. 5). One notable fact is that most of them did not even beat the baseline method logistic regression with linear kernel function. Therefore, traditional TL techniques such as sample weighting and feature alignment strategies may not be effective for us to delineate the distribution patterns of L-MCI and E-MCI. Since our method compared distributions directly, we could glean more information from AD and NC patients as well as MCI patients in the training set. We also found that Easy MKL, average KL, and PWMK methods yielded relatively high performance on both domains. We concluded that combining multiple kernel functions in an appropriate manner can improve the classification performance.

### Bootstrap aggregation comparison results

**Fig. 6:**
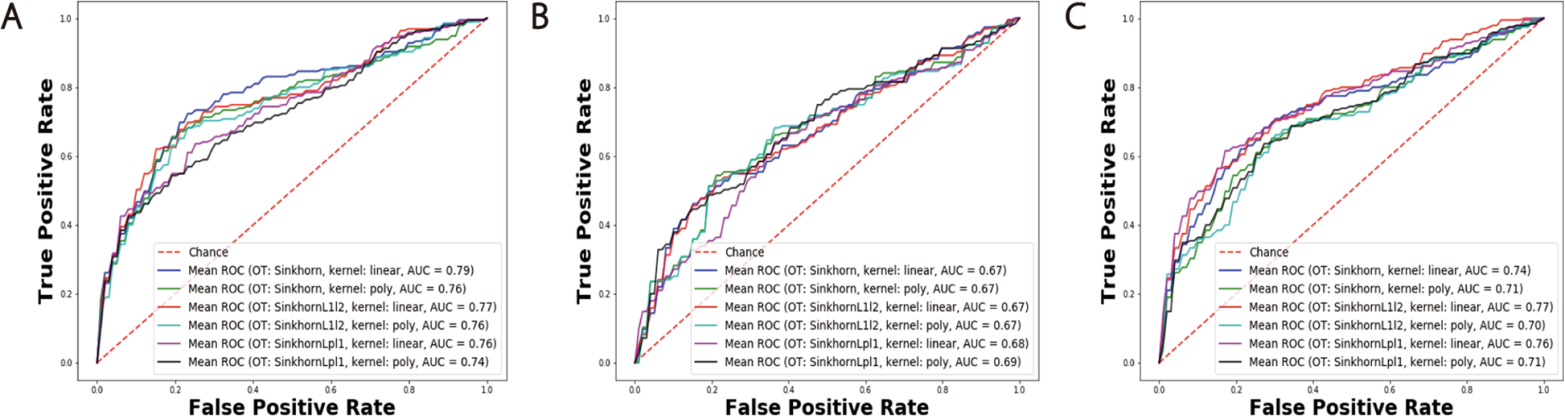
AUC curves of BAg of our OT transfer learning framework. Panels **A**, **B**, and **C** correspond to results on the FDG, VBM, and combination of two data modalities respectively. The highest AUC curve is achieved by using SD combined with *L*_1_*l*_2_ regularization term as OT mapping cost function and linear kernel logistic regression as classifier

**Fig. 7:**
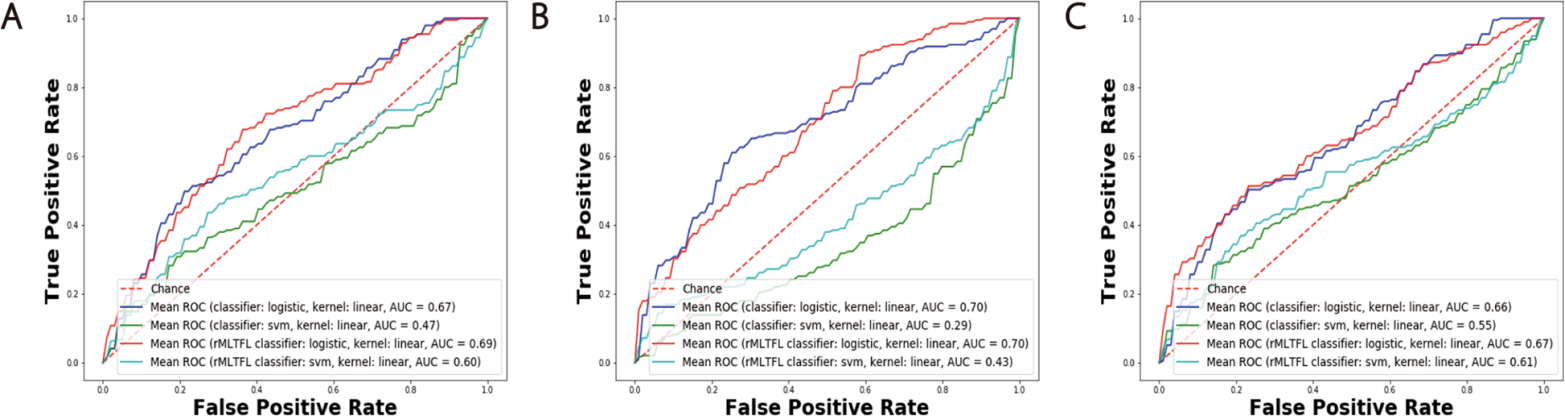
AUC curves of BAg of logistic regression, SVM, and rMLTFL (logsitic regression and SVM as classifier). Panels **A**, **B**, and **C** correspond to results on the FDG, VBM, and combination of two data modalities respectively

In Table 4, we list the aggregated model performance of the testing set for different models and different data modalities. Besides our OT mapping strategies, we also implemented the BAg using two baseline methods and the rMLTFL benchmark method. The performance of our model was significantly superior than SVM, logistic regression, and rMLTFL (Figs. 6 and 7). By choosing different training sets, our model captured heterogeneous patterns. When we aggregated them using a voting strategy, most models could correctly prediction the testing samples. Hence, the accuracy as well as AUC score was much higher than the single model case. On the other hand, the logistic regression, SVM, and rMLTFL models were quite stable with regard to the training set (Figs. 6 and 7). The patterns they learned are quite homogeneous. We conclude that learning sub-models does not improve model performance for these baseline and benchmark methods.

**Table 4:** Accuracy (ACC) and AUC score of BAg results

Methods	FDG		VBM		FDG + VBM	
	ACC	AUC	ACC	AUC	ACC	AUC
SD/l	72.82	0.79	68.48	0.67	70.65	0.74
SD/p	73.91	0.76	64.13	0.67	69.56	0.71
SD *L*_1_*l*_2_/l	**75.00**	**0.77**	67.39	0.67	69.56	0.77
SD *L*_1_*l*_2_/p	71.74	0.76	63.04	0.67	65.21	0.70
SD *L*_*p*_*l*_1_/l	71.74	0.76	59.78	0.68	73.91	0.76
SD *L*_*p*_*l*_1_/p	71.74	0.74	67.39	0.69	66.30	0.71
SVM	57.61	0.47	57.61	0.29	57.61	0.55
logistic	68.47	0.67	58.70	0.70	67.39	0.66
rMLTFL	63.04	0.69	60.87	0.70	63.04	0.67

The OT method and kernel function combination is denoted as OT method/kernel function. l and p represent linear and polynomial kernel respectively

**Fig. 8:**
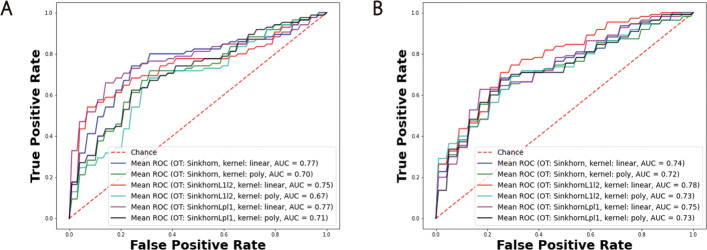
AUC curves of BAg of logistic regression, SVM, and rMLTFL (logsitic regression and SVM as classifier). Panels **A** and **B**, correspond to results on the validation and testing datasets respectively

In order to evaluate the reproducibility of our proposed method, we further split the dataset into three subsets, namely training (80*%*), validation (10*%*), and testing (10*%*) datasets. By training and aggregating models based on training samples and testing on the validation and testing datasets, we obtain AUC curves in Fig. 8. It implies that our model can yield plausible and stable results (highest AUC score = 0.77 on the validation dataset and 0.78 on the testing dataset) regardless how do we split the dataset. Due to the limitation of ADNI patient labels, we cannot perform experiments on other interesting tasks such as P-MCI versus S-MCI. But we added two more experiments on AD vs MCI and MCI vs NC to illustrate the effectiveness of our framework. We demonstrate our results and benchmark studies in the supplementary material (Table S1 and S2).

## Discussion

We present our novel method which uses optimal transport to improve the performance discriminating between (E-MCI vs L-MCI) using MRI and PET images. We found that by using OT theory to project the more difficult task, E-MCI vs L-MCI, onto the easier task of distinguishing AD and NC, we were able to achieve higher performance than by using MCI samples alone. This represents not only a significant advance in OT and TL methods but also has clear clinical implications.

Indeed, identifying cognitively impaired individuals early will likely their health outcomes because of early access to treatment and monitoring [33, 34]. These early detection systems are most frequently focused on the readily available and minimally invasive medical imaging procedures like MRI and PET scans. Ideally, at risk patients could regularly be tested for AD and MCI by their physicians. These imaging technologies offer a potential avenue to a minimally invasive test for cognitive impairment. These clinical tests however are dependent on accurate ML models which can effectively discriminate between cognitively normal, end stage Alzheimers, and the entire spectrum in between.

By using OT to map E-MCI and L-MCI samples to the auxiliary domain, we reduce the inter-task discrepancy between AD vs NC task and E-MCI vs L-MCI task while maximizing the intra-task differences. This TL technique enable us to train LR classifiers which can stratify E-MCI and L-MCI patients more accurately. We then aggregate sub-TL models using a majority voting strategy to improve the model stability and avoid the overfitting issue.

With the novel methods that we have developed, we outperform the current state-of-the-art TL methods and show that it is crucial to leverage AD and NC data to accurately predict L-MCI and E-MCI patients. Such continued improvements are necessary to improve the personal, healthcare, and economic costs [35] associated with over six million AD patients in the USA alone.

## Limitations

When compared with other benchmark works, our model yields a high prediction accuracy and AUC score. We also acknowledge several limitations. Our feature selection method rMLTFL depends on three hyper-parameters. It’s of crucial importance to select correct combination hyper-parameters. Although we grid search them over 1000 combinations, there is still lack of evidence that the selected combination is an optimal choice. Furthermore, we have not considered its performance in other challenging MCI classification tasks such as the P-MCI and S-MCI classification task [13]. Finally, our framework is developed based on the VBM and FDG features extracted in [18], which have been exploited in some related studies. Currently the reported performances of all these studies are not good enough for clinical treatment. Potential strategies for improving stratification performance include (1) using more samples for training when more samples are available in the ADNI dataset; (2) since VBM and FDG features extracted by [18] may not be representative enough for distinguishing different MCI conditions, we could try to adopt more advance feature extracting pipelines; and (3) combining image features with genotype profiles for more accurate assessment. Since genotype data may provide supplementary information to image data, we could train more accurate and stable models based on combining these two heterogeneous data modalities.

## Conclusion

We have developed an optimal transport based transfer learning model to discriminate between E-MCI and L-MCI patients. Our methods are both novel and the current state of the art based on benchmark comparisons. This method is a necessary technological stepping stone to widespread clinical usage of MRI based early detection of AD.

## Supplementary Information

**Additional file 1** This csv file contains the pre-processed FDG features from [18]. Label 1, 3, 4, 5 correspond to NC, E-MCI, L-MCI and AD subjects respectively.

**Additional file 2** This csv file contains the pre-processed VBM features from [18]. Label 1, 3, 4, 5 correspond to NC, E-MCI, L-MCI and AD subjects respectively.

**Additional file 3** This pdf file contains two tables which include results on two related tasks (table 1, 2).

## Data Availability

The dataset(s) supporting the conclusions of this article is(are) included within the article (and its additional file(s).

## Abbreviations

AD: Alzheimer’s disease
MCI: Mild cognitive impairment
NC: Normal control
L-MCI: Late stage mild cognitive impairment
E-MCI; Early stage mild cognitive impairment; CT: Computed tomography
MRI: Magnetic resonance imaging
PET: Positron emission tomography
VBM: Voxel-based measure
FDG: fluorodeoxyglucosee TL: Transfer learning
ADNI: The Alzheimer’s Disease Neuroimaging Initiative
MMSE: Mini-Mental State Examination
CDR: Clinical dementia rating
BAg: Bootstrap aggregation
OT: Optimal transport
rMLTFL: Multi-label transfer feature learning
SVM: Support vector machine
LR: Logistic regression
ANOVA: Analysis of variance
t-SNE: t-distributed stochastic neighbor embedding
SD: Sinkhorn distance
AUC: Area under the receiver operating curve
IW: Importance-weighting
TCA: Transfer component analysis
SUBA: Semi-supervised subspace alignment
FLDA: Feature-level domain adaptation
TrAdaBoost: Boosting for transfer learning
MKL: Multiple kernel learning
AverageMKL: sSmple average of base kernels
EasyMKL: Radius-based combination of kernels
GRAM: Radius-margin ratio optimization for dot-product boolean kernel learning
RMKL: Radius-based multiple kernel learning
PWMK: Simple but effective methods for combining kernels in computational biology
CKA: Centered kernel alignment optimization in closed form
