# Supplementary material for: Optimal transport- and kernel-based early detection of mild cognitive impairment patients based on magnetic resonance and positron emission tomography images
Source: Alzheimers Res Ther. 2022 Jan 7;14:4. doi: 10.1186/s13195-021-00915-3 (PMC8742368; doi:10.1186/s13195-021-00915-3)
Supplement: Supplementary file 3 — Additional file 3 This pdf file contains two tables which include results on two related tasks (table 1, 2). [file 13195_2021_915_MOESM3_ESM.pdf]

RESEARCH

Optimal transport- and kernel-based early detection of mild cognitive impairment patients based on magnetic resonance and positron emission tomography images

Ziyu Liu[1], Travis S. Johnson[2], Wei Shao[2], Min Zhang[1], Jie Zhang[4*] and Kun Huang[2,3*]

Table 1 Accuracy and AUC score of baseline, transfer learning and Multi-kernel benchmark methods on the AD versus MCI task. For benchmark methods which don't produce the AUC score or don't converge, we denote their scores by slash lines.

Methods	Accuracy	AUC
SVM	87.71	86.00
Logistic Reg	89.47	86.57
rMLTFL	89.47	86.57
IW	91.22	89.71
TCA	87.71	/
SUBA	87.71	81.43
RBA	87.71	0.50
FLDA	/	/
TrAdaBoost	87.72	/
Easy MKL	91.22	/
Average MKL	91.22	/
PWMK	91.22	/
GRAM	91.22	/
RMKL	89.47	/
CKA	87.72	/
Our Framework	91.22	84.57

Table 2 Accuracy and AUC score of baseline, transfer learning and Multi-kernel benchmark methods on the MCI versus NC task. For benchmark methods which don't produce the AUC score or don't converge, we denote their scores by slash lines.

Methods	Accuracy	AUC
SVM	81.92	88.09
Logistic Reg	82.46	88.30
rMLTFL	82.46	88.30
IW	84.21	93.62
TCA	81.47	/
SUBA	80.70	78.79
RBA	81.48	50.00
FLDA	/	/
TrAdaBoost	84.12	/
Easy MKL	76.67	/
Average MKL	76.67	/
PWMK	76.67	/
GRAM	76.67	/
RMKL	76.67	/
CKA	76.67	/
Our Framework	81.97	70.19

From table 1 and 2 we observe that although our proposed method doesn't achieve the best performance on two tasks, its performance is stable across different tasks. For instance, multi-kernel methods outperform all benchmark methods on the AD versus MCI task, but their performances are poor on the MCI versus NC task. Therefore, our method produces more stable results.

Author details
[1]Department of Statistics, Purdue University, West Lafayette, USA.
[2]Biostatistics and Health Data Science, Indiana University School of Medicine, Indianapolis, USA. [3]Regenstrief Institute, Indianapolis, USA.
[4]Department of Medical and Molecular Genetics, Indiana University School of Medicine, Indianapolis, USA.

References

*Correspondence: jizhan@iu.edu; kunhuang@iu.edu
[4]Department of Medical and Molecular Genetics, Indiana University School of Medicine, Indianapolis, USA
[2]Biostatistics and Health Data Science, Indiana University School of Medicine, Indianapolis, USA
[3]Regenstrief Institute, Indianapolis, USA
Full list of author information is available at the end of the article
